# Long-term survival and prognostic implications of patients with invasive breast cancer in southern Taiwan

**DOI:** 10.1097/MD.0000000000019122

**Published:** 2020-02-14

**Authors:** Shih-Chung Wu, Ming-Chu Chiang, Yun-Gang Lee, Mei-Wen Wang, Chuan-Fang Li, Tao-Hsin Tung, Hsiao-Hui Chen

**Affiliations:** a Department of General Surgery; b Department of Nursing; c Cancer Canter, Kaohsiung Chang Gung Memorial Hospital and Chang Gung University College of Medicine, Kaohsiung; d Department of Medical Research and Education, Cheng Hsin General Hospital, Taipei, Taiwan.

**Keywords:** women, invasive breast cancer, prognostic factor, survival

## Abstract

Our objective in this study was to determine the survival rate of patients with invasive breast cancer and identify the prognostic factors related to all-cause mortality during a 10-year follow-up.

Analysis was performed on the medical records of 2002 patients newly diagnosed with breast cancer at a medical center in southern Taiwan between 2006 and 2017. The Kaplan–Meier method and Cox regression analysis were used to estimate survival and the independence of prognostic factors associated with all-cause mortality.

Among the 2002 patients, 257 expired during the 10-year follow-up period. The overall survival rates were as follows: 3 years (91.1%), 5 years (85.6%), and 10 years (77.9%). The median survival time was 120.41 months (95% confidence interval: 118.48–122.33 months). Older age, pathologic tumor status, regional lymph node metastasis, distant metastasis, grade/differentiation, treatment modalities, and hormone therapy were significantly related to all-cause mortality.

This study identified several clinical factors related to all-cause mortality as well as its relationship to distant metastasis and poor differentiation. Early diagnosis and treatment aimed at preventing recurrence are the keys to survival.

## 1 Introduction

Breast cancer is the leading cause of cancer-related death among women worldwide. A 50-year-old woman without cancer has a 2.3% risk of developing breast cancer during the next 10 years (i.e., 1 in every 43 women will be diagnosed with breast cancer by the age of 60 years).^[1]^ In recent decades, there has been a continual increase in the incidence of breast cancer, particularly in low- and middle-income countries. Note that long-term survival in countries with modern health care can reach 80% to 85%^[2,3]^; however, there is considerable variation in these outcomes. The 5-year survival rate is very high in Australia (89.5%) and the United States (90.2%), but it is quite low in developing countries such as India (66.1%).^[4]^

In Taiwan, malignant tumors have been the most common cause of death since 1982. Nonylphenol intake has been linked to the incidence of breast cancer in Taiwan.^[5]^ In fact, the average daily intake of nonylphenol in Taiwan is 4× higher than in Germany and 8.5× higher than in New Zealand. Within the group of estrogen receptor + (ER+) tumors is the ER+/pathologic response – (PR–) subtype associated with less favorable outcomes.^[6]^ The etiology of breast cancer has generally been attributed to genetic, reproductive, and hormonal factors. In 1 study in Taiwan, estrogen-related factors, such as obesity, endometriosis, uterine myoma, hypertension, and dyslipidemia, were identified as important risk factors for breast cancer.^[7]^ Other factors linked to the prognosis and survival of patients include tumor stage, tumor size, tissue morphology, degree of differentiation, and patient age.^[8]^

There have been relatively few studies on the long-term prognosis of patients with invasive breast cancer in southern Taiwan. This study sought to fill this gap using data from cancer databases from a medical center in that region. Analysis was conducted on sociodemographic and clinicopathologic characteristics as well as survival rates and factors affecting survival for the periods of 3, 5, and 10 years after diagnosis.

## 2 Methods

### 2.1 Patient population

**Figure 1:**
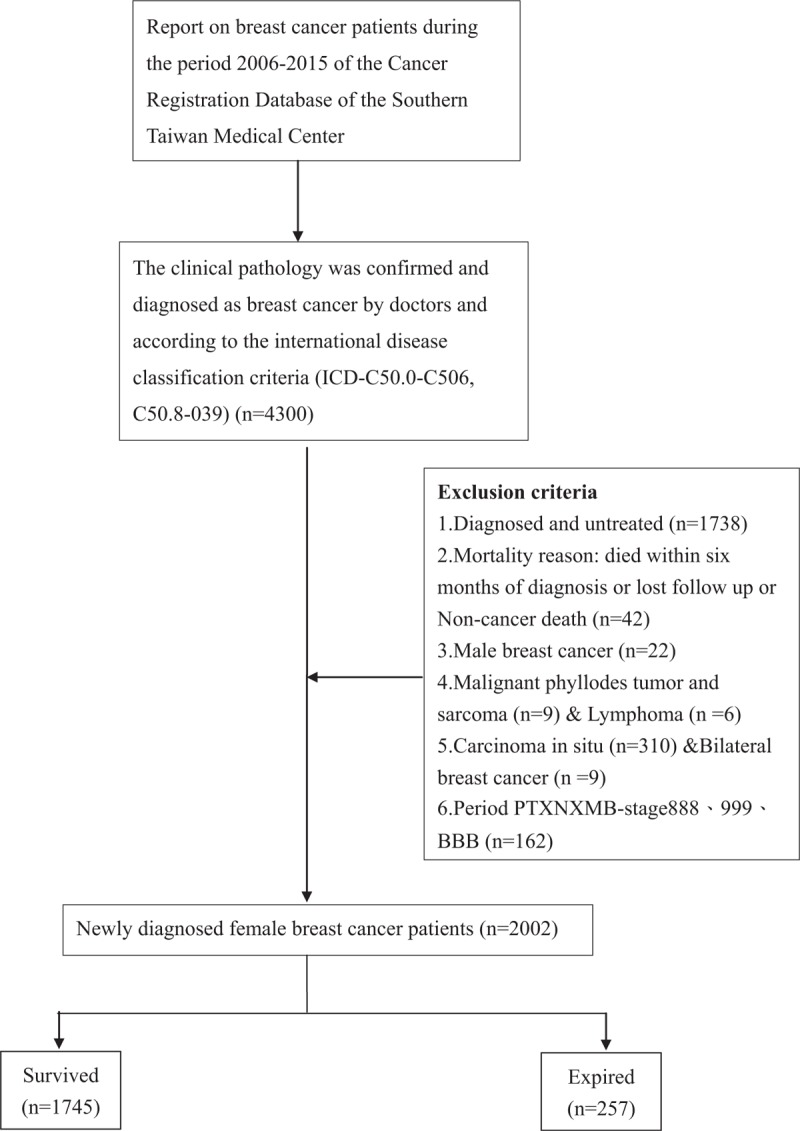
Selection of the study subjects.

As shown in Figure 1, a total of 4300 patients with invasive breast cancer were identified at the Cancer Center of Kaohsiung Chang Gung Memorial Hospital between January 2006 and June 2017. Assessments were based on the 6th and 7th editions of the pathologic staging criteria formulated in 2010 by the American Joint Committee on Cancer (AJCC) in accordance with pathologic T, pathologic N, and pathologic M tumor/lymph node/metastasis (TNM). Tumor prognosis was linked to the size of the primary tumor and characteristics of the surrounding structures as well as the number and/or location of regional lymph nodes, the presence/ absence of extracapsular extension, and the presence/absence of distant metastasis.^[5,9]^ Of the 4300 patients with breast cancer identified in the study period, 2298 (53.4%) were excluded due to the presence of lymphoma, death within 6 months, or diagnosis in other hospitals (i.e., inability to confirm diagnostic parameters). The remaining 2002 patients with breast cancer were evaluated in this study.

### 2.2 Data collection

Patient baseline characteristics and surgical variables were retrieved from medical records and computer files. The hospital records of each patient were reviewed by well-trained, senior medical chart reviewers using a standard data collection form. Demographic information, the presence of preexisting comorbidities, and medications prescribed at the time of admission and discharge were obtained from nursing and medical histories. In the International Classification of Cancer, T refers to the size and extent of the main tumor, otherwise referred to as the primary tumor; N refers to the extension; and M indicates whether the cancer has metastasized (i.e., spread from the primary tumor to other parts of the body).^[11]^ Access to hospital records was approved by the Human Subjects Review Board at Kaohsiung Chang Gung Memorial Hospital (no: 201700844B0C601).

### 2.3 Statistical analysis

Statistical analysis was performed using SPSS 23.0. Prognostic predictors and all-cause mortality were determined using univariate as well as multivariate techniques. In univariate analysis, the Chi-squared test was used for discrete variables and a 2-sample independent Student *t* test was used for continuous variables. The Kaplan–Meier method with log-rank test was used to estimate the cumulative survival of patients with breast cancer. Multiple Cox regression was used to investigate the independence of factors associated with all-cause mortality based on variables identified in univariate analysis. Subjects were considered censored if the outcomes were unavailable. A *P*-value of <.05 was considered statistically significant. The results are presented as the mean ± standard deviation.

## 3 Results

Table 1 lists the baseline characteristics of patients with breast cancer included in the study. The patients were divided into 5 age groups as follows: <40 years (n = 156; 7.8%), 40 to 49 years (n = 537; 26.8%), 50 to 59 years (n = 716; 35.8%), 60 to 69 years (n = 407; 20.3%), and ≥70 years (n = 186; 9.3%). The overall staging results at the time of diagnosis were as follows: tumor stage I or II (78.5%), pathologic tumor status (T1) (n = 946; 47.3%), pathologic node status (N0) (n = 1243; 62.1%), distant metastasis (n = 74; 3.7%), moderate differentiation (n = 900; 45.0%), tumor size ≥2 to 5 cm (n = 958; 47.9%), and single site (n = 1555; 78.0%).

**Table 1:**
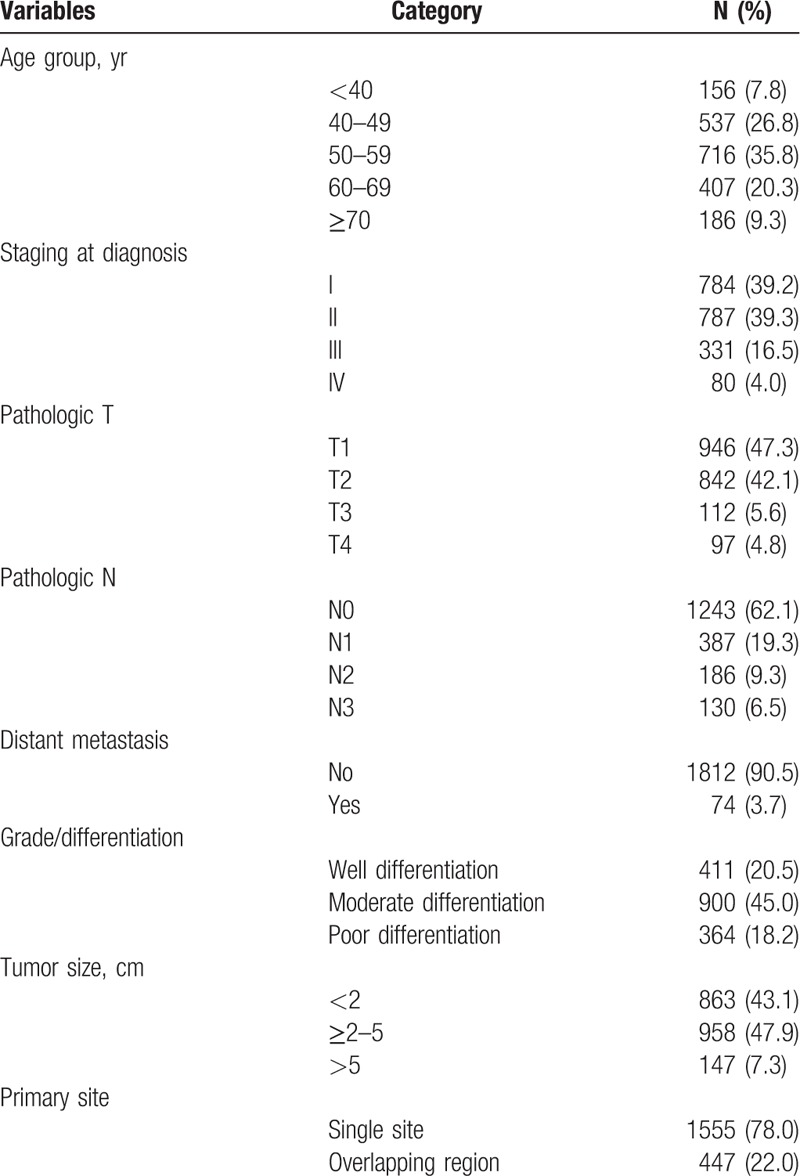
Different stages of breast cancer according to the AJCC classification (n = 2002).

Among the 2002 patients included in the study, 257 expired within 10 years following diagnosis. As shown in Figure 2 A, the cumulative survival was as follows: 3-year (91.1%), 5-year (85.63%), and 10-year (77.9%). The statistical significance for 10-year cumulative survival was as follows: stage (*P* = .0001), pathologic T status (*P* = .0003), pathologic N status (*P* = .0001), distant metastasis (*P* = .0001), grade differentiation status (*P* = .0001), tumor size (*P* = .0001), and primary-site status (*P* = .003).

**Figure 2:**
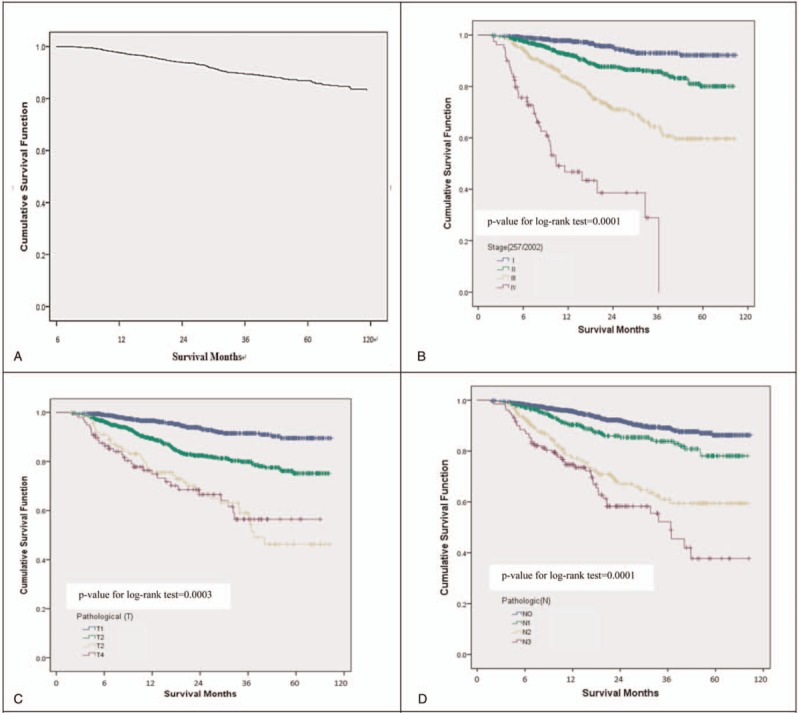
The overall cumulative survival rate for patients with breast cancer (A), and stratified by stage (B), pathologic T status (C), pathologic N Status (D), distant metastasis (E), grade differentiation status (F), tumor size (G), and primary-site status (H).

**Figure 2 (Continued):**
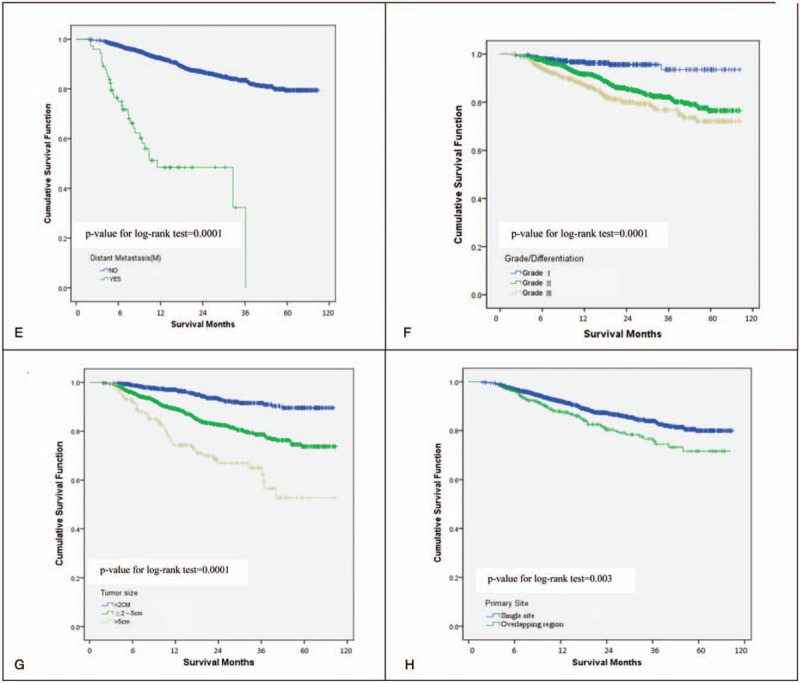
The overall cumulative survival rate for patients with breast cancer (A), and stratified by stage (B), pathologic T status (C), pathologic N Status (D), distant metastasis (E), grade differentiation status (F), tumor size (G), and primary-site status (H).

The multiple Cox regression model was used to examine the influence of independently associated risk factors on all-cause mortality, the results of which are listed in Table 2. After adjusting for confounding factors, the following items were significantly related to all-cause mortality: age (≥70 years vs <40 years, hazard ratio [HR]: 2.26, 95% confidence interval [CI]: 1.16–4.43), pathologic T (T2 vs T1 = HR: 1.53, 95% CI: 1.04–2.26; T3 vs T1 = HR: 1.96, 95% CI: 1.15–3.35; T4 vs T1 = HR: 2.62, 95% CI: 1.49–4.63), pathologic N (PN2 vs PN0 = HR: 2.40, 95% CI: 1.60–3.58; PN3 vs PN0 = HR: 2.76, 95% CI: 1.76–4.33), distant metastasis (yes vs no = HR: 3.51, 95% CI: 1.98–6.23), grade differentiation (poor vs well = HR: 1.81, 95% CI: 1.00–3.29), and hormone therapy (yes vs no = HR: 0.41, 95% CI: 0.30–0.55).

**Table 2:**
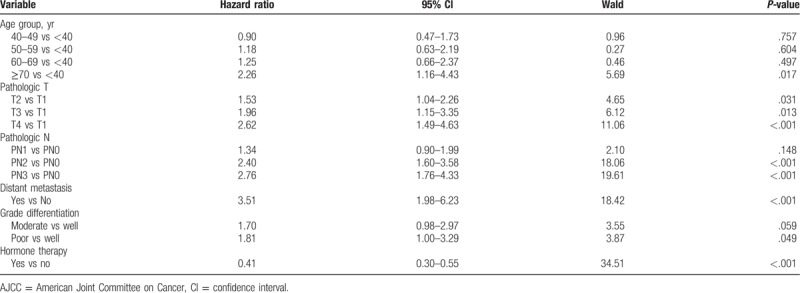
Multivariate analysis using Cox regression model of risk factors associated with the all-cause mortality that all univariate significant factors were included among patients with breast cancer (n = 2002).

## 4 Discussion

### 4.1 Risk factors for all-cause mortality: implications

The enormous financial burden imposed by breast cancer (in terms of treatment, nursing, non-medical expenses, and losses in productivity) is expected to continue increasing well into the future. Consensus guidelines for the diagnosis and treatment of breast cancer issued by the Taiwan Cooperative Oncology Group (TCOG) stipulate that tumor staging and prognosis be used to determine the most appropriate treatment regime (i.e., surgery, chemotherapy, hormonal therapy, or radiotherapy).^[10]^ In this study, we estimated the overall survival of patients newly diagnosed with breast cancer at a medical center in southern Taiwan (phases I–IV), as follows: 5 years (85.6%) and 10 years (77.9%). The overall 5-year survival rates in other countries were as follows: India (46%),^[11]^ Oman (64%),^[12]^ Greece (65%),^[13]^ Germany (71%),^[14]^ the United States (89%),^[15]^ and the United Kingdom (84%).^[15]^ Clearly, the long-term survival of patients with breast cancer in southern Taiwan exceeds that of most parts of the world.

From an epidemiologic perspective, the cancer incidence is largely age dependent, wherein 50% of all patients with breast cancer occur in women between the ages of 50 and 69 years.^[16]^ In this study, 38% of the patients were younger than 50 years, and the HR of patients who developed breast cancer at an age of ≥50 years relative to those who developed breast cancer at an age of ≤50 was 1.56 (95% CI: 1.11–2.21). This is a clear indication that advanced age was associated with an increase in the risk of mortality, which is consistent with results in previous studies.^[17]^

The factors with the most pronounced impact on survival were lymph node invasion (N), tumor size (T), distant metastasis (M), and vascular invasion of the lymph nodes. Patients with poorly differentiated tumor faced a risk of death double that of patients with well-differentiated tumor, which is in line with results of previous studies.^[18]^ Since 2002, the Ministry of Health and Welfare in Taiwan has conducted a national biennial mammography screening program for women aged between 40 and 69 years.^[19]^ They reported that 690,000 women underwent mammography screening in 2013, which represents a screening rate of 36%.^[20]^ It has been reported that population-based mammography screening is associated with a 41% reduction in breast cancer mortality rates.^[21]^

### 4.2 Clinical practice

Current recommendations to avoid breast cancer include avoiding exposure to plasticizers in many products, using environmentally friendly cups, and engaging in regular exercise. It is also recommended that all women undergo regular screening tests (e.g., breast self-examination, breast ultrasonography, or mammography), and a number of researchers have described how campaigns on social media can be used to educate the populace as to the importance of early detection and early treatment. Tumor size can be used to gauge the response to chemotherapy, thereby decreasing the need for breast resection, particularly among patients below the age of 35. Newly developed treatment combinations are also expected to reduce the rate of recurrence and improve survival rates.

### 4.3 Methodologic considerations

This prospective study was based on a long-term follow-up of all patients with invasive breast cancer in a well-defined population; however, there are a number of limitations that must be taken into account. It was not possible to avoid potential Berkson bias (selection bias) due to the hospital-based design of the study. The fact that this group of patients cannot be precisely representative of the general population makes it difficult to estimate the long-term survival of age-matched population in southern Taiwan. Second, some of the clinical factors derived from medical records were prone to misclassification bias. Nevertheless, it seems reasonable to assume that misclassification bias was not associated with all-cause mortality and could therefore be viewed as nondifferential misclassification. Third, our inability to obtain quantitative information pertaining to many potential risk factors and lifestyle habits (e.g., exercise) made it impossible to clarify the dose-response effect between personal habits and all-cause mortality. Finally, this study dealt exclusively with patients from one medical center in southern Taiwan; therefore, our results cannot be extrapolated to hospitals in other regions of Taiwan. Future research covering hospitals in other regions would make the findings more discursive.

## 5 Conclusion

This study sought to identify the relationship between all-cause mortality among patients with invasive breast cancer and patient age, pathologic staging, distant metastasis, and hormone therapy. Early diagnosis and treatment aimed at preventing recurrence are the keys to survival.

## Acknowledgment

The authors thank the Sunflower Statistical Consulting Company, Kaohsiung, Taiwan for statistical advice.

## Author contributions

**Formal analysis:** Hsiao-Hui Chen, Yun-Gang Lee.

**Methodology:** Shih-Chung Wu, Ming-Chu Chiang.

**Writing – original draft:** Shih-Chung Wu, Hsiao-Hui Chen, Mei-Wen Wang, Chuan-Fang Li.

**Writing – review & editing:** Tao-Hsin Tung.
